# Mouse Models for HTLV-1 Infection and Adult T Cell Leukemia

**DOI:** 10.3390/ijms241411737

**Published:** 2023-07-21

**Authors:** Shinsuke Nakajima, Kazu Okuma

**Affiliations:** Department of Microbiology, Faculty of Medicine, Kansai Medical University, Hirakata 573-1010, Osaka, Japan; nakajims@hirakata.kmu.ac.jp

**Keywords:** HTLV-1, ATL, Tax, HBZ, immunodeficient mouse, humanized mouse

## Abstract

Adult T cell leukemia (ATL) is an aggressive hematologic disease caused by human T cell leukemia virus type 1 (HTLV-1) infection. Various animal models of HTLV-1 infection/ATL have been established to elucidate the pathogenesis of ATL and develop appropriate treatments. For analyses employing murine models, transgenic and immunodeficient mice are used because of the low infectivity of HTLV-1 in mice. Each mouse model has different characteristics that must be considered before use for different HTLV-1 research purposes. HTLV-1 *Tax* and *HBZ* transgenic mice spontaneously develop tumors, and the roles of both Tax and HBZ in cell transformation and tumor growth have been established. Severely immunodeficient mice were able to be engrafted with ATL cell lines and have been used in preclinical studies of candidate molecules for the treatment of ATL. HTLV-1-infected humanized mice with an established human immune system are a suitable model to characterize cells in the early stages of HTLV-1 infection. This review outlines the characteristics of mouse models of HTLV-1 infection/ATL and describes progress made in elucidating the pathogenesis of ATL and developing related therapies using these mice.

## 1. Introduction

Human T cell leukemia virus type 1 (HTLV-1) was first discovered as a pathogenic human retrovirus [1,2]. At least 5 to 10 million people worldwide are infected with HTLV-1, and regions with large numbers of HTLV-1 carriers include Japan, the Caribbean Islands, Latin America, regions of tropical Africa, and Central Australia [3]. HTLV-1 primarily infects CD4^+^ T cells, and the virus is not eliminated and persists throughout life. Although most infected individuals are asymptomatic, HTLV-1 can cause malignant tumors known as adult T cell leukemia (ATL) [4,5] and neurodegenerative diseases such as HTLV-1-associated myelopathy (HAM) or tropical spastic paraparesis (TSP) after a latent period of several years to several decades [6]. The lifetime risk of developing ATL is 6–7% in HTLV-1-infected men and 2–3% in HTLV-1-infected women in Japan [7], while the risk of developing HAM/TSP is approximately 0.25–3.8% in HTLV-1-infected individuals [8]. Although new treatments for these diseases are being developed, they remain refractory.

ATL is classified into four disease types: smoldering, chronic, acute, and lymphoma [9]. When ATL progresses to acute or lymphoma types, the 4-year survival rate is approximately 10% [10]. Acute ATL is characterized by increased lymphocytes and eosinophils, the detection of abnormal lymphocytes (called flower cells), hepatosplenomegaly, lymphadenopathy, bone marrow infiltrates, and osteolytic lesions, resulting in hypercalcemia, and skin lesions. The HTLV-1 proteins Tax and HBZ are thought to be involved in the pathogenesis of ATL. Tax promotes viral transcription by activating the 5′ long terminal repeat (LTR). In addition, Tax transforms cells and can induce cell proliferation and immortalization. In vitro, Tax induces immortalization when transfected into T cells and fibroblasts. In vivo, Tax transgenic mice develop tumors. Tax activates nuclear factor (NF)-κB, nuclear factor of activated T cells (NFAT), and activator protein 1 (AP-1) pathways, suppresses p53 function, and regulates various intracellular signaling. Constitutive activation of NF-κB by Tax is important for cell transformation and tumor growth. In vivo, Tax is rarely expressed in HTLV-1-infected cells because of its high immunogenicity. In vitro experiments suggest that the expression pattern of Tax is transient [11], making it possible that transient expression of Tax in vivo allows cells to evade attacks from cytotoxic T cells (CTLs). Half of the patients with ATL are unable to fully express Tax due to the loss of the 5′ LTR or loss of transcriptional activity via methylation [12,13]. These findings suggest that Tax is not essential for the survival of infected cells with oncogenic transformation (ATL cells). *HBZ* is a gene encoded on the minus strand. Unlike Tax, HBZ is less immunogenic and is constitutively expressed in HTLV-1-infected cells and cancer cells. HBZ transgenic mice develop tumors [14]. Moreover, HBZ promotes ATL cell proliferation [15], inhibits apoptosis [16], and modulates DNA damage responses [17].

Treatments for ATL include multidrug chemotherapy, zidovudine/interferon (IFN) combination therapy [18,19], and allogeneic hematopoietic stem cell transplantation [20]. In addition, treatment with mogamulizumab, an anti-CCR4 antibody, has shown some efficacy in the treatment of ATL [21,22]. Tax peptide-pulsed dendritic cell vaccine (Tax-DC), which enhances Tax-specific CTL responses, has also shown efficacy against ATL in clinical trials [23].

ATL cell lines and peripheral blood mononuclear cells (PBMCs) derived from patients with ATL have been used for in vitro screening of ATL therapeutics. When PBMCs derived from patients with ATL are cultured ex vivo, the expression of Tax is induced. However, cells activated by Tax in vitro or ex vivo may not be consistent with the cellular state of cells activated in vivo. Therefore, the development of therapeutic agents for ATL requires animal models that can evaluate drug efficacy in vivo. Various animal models have been used to elucidate the pathogenesis of ATL and develop relevant therapies. Old World monkeys and apes, including Japanese monkeys and Cynomolgus monkeys, are naturally infected with STLV-1, which is closely related to HTLV-1 [24]. STLV-1-infected monkey models have been used to study vaccines and therapeutics for ATL [25,26,27,28]. HTLV-1-infected monkey models have also been used to study the mechanisms of infection and immune responses during the early stages of HTLV-1 infection [29,30]. The disadvantage of studies using non-human primates is their high cost. Mice entail low costs and are easy to maintain, but HTLV-1 does not infect mice efficiently. Because mice are easy to genetically modify, transgenic and severely immunodeficient mice have been generated and used for HTLV-1 research. Each mouse model has different characteristics and is used to for the purpose of HTLV-1 research. Transgenic mouse models have greatly contributed to the analysis of Tax and HBZ in the transformation of HTLV-1-infected cells and tumor growth. Severely immunodeficient mice can be engrafted with ATL cell lines, and an ATL model was developed to investigate therapeutic agents. Humanized mice with a human immune system can be characterized during the early stages of HTLV-1 infection. Currently available humanized mice have insufficiently reconstructed human immune cells and cannot be used for experiments examining human immune responses, such as antibody production and cytotoxic activity. However, next-generation humanized mice with improved reconstitution of human immune cells are now under development. In the future, the use of humanized mice with acquired immunity in addition to innate immunity will enhance HTLV-1 research, including the development of HTLV-1 vaccines. Here, we introduce the characteristics of each mouse model of HTLV-1 infection and ATL, as well as findings obtained using these mice.

## 2. Transgenic Mouse Models for HTLV-1 Research

To investigate the role of HTLV-1 in the pathogenesis of ATL, Tax and HBZ transgenic mice were generated. HTLV-1 mainly infects CD4^+^ T cells in humans, but Tax transgenic mice express Tax in different cells and tissues depending on the promoter used for gene expression. Transgenic mice in which the *Tax* gene is under the control of the LTR promoter (LTR-Tax) express Tax specifically in tissues such as the thymus and muscle, and exhibit mesenchymal tumors [31]. Although LTR-Tax transgenic mice do not display ATL-like signs such as lymphoma or leukemia, Tax was proven to have a transformative activity and cause tumor formation. Some transgenic mice also developed thymic atrophy [32], neurofibromas composed of perineural fibroblasts [33], exocrine abnormalities involving the salivary and lacrimal glands (similar to Sjögren’s syndrome) [34], chronic arthritis similar to rheumatoid arthritis [35,36,37], and myelofibrosis [38]. To investigate Tax expression in detail within tissues in vivo, double-transgenic mice were generated by crossing LTR-Tax transgenic mice with LTR-βgal transgenic mice. These mice express Tax in a tissue-specific manner in muscle, bone, salivary glands, skin, and nerves [39]. Subsequently, transgenic mice have been generated using various promoters to express Tax specifically in lymphocytes. Transgenic mice expressing Tax under the control of the human granzyme B promoter (GzmB-Tax) develop large granular lymphocytic (LGL) leukemia [40]. LGL cell lines established from GzmB-Tax transgenic mice exhibit pre-NK cell surface markers [41]. GzmB-Tax transgenic mice were observed to have hypercalcemia and osteolytic bone metastases, which frequently occur in patients with ATL [42]. Transgenic mice expressing Tax under the control of the CD3-epsilon promoter present generation of mesenchymal tumors, as well as salivary and mammary adenomas [43]. Transgenic mice expressing Tax under the control of the proximal promoter of Lck (Lck-Tax), which restricts Tax expression to thymocytes, develop diffuse large cell lymphoma and leukemia after a long latent period. The phenotypes of lymphoma cells from Lck-Tax transgenic mice, which were transferred into severe combined immunodeficiency (SCID) mice, were pre-T cells (CD4^−^/CD8^−^/CD44^+^/CD25^+^ and cytosolic CD3^+^) [44]. Lck-distal Tax transgenic mice expressing Tax in mature thymocytes and peripheral T lymphocytes were generated. These mice develop T cell leukemia and lymphoma, with the major phenotype of leukemic cells being mature CD4^+^ or CD8^+^ T cells [45]. In summary, Tax was shown to have cell-transforming activity and cause tumors and various clinical manifestations. When Tax expression is restricted to lymphocytes, mice develop leukemias and lymphomas, showing ATL-like clinical signs such as hypercalcemia and osteolytic bone metastases.

The characteristics of tumors and tissues transformed by Tax in Tax transgenic mice have been analyzed. Fibroblast tumors generated in Tax transgenic mice express high levels of granulocyte–macrophage colony-stimulating factor, interleukin (IL)-6, platelet-derived growth factor β, Zif268, and c-fos [46]. Transforming growth factor (TGF)-β1 mRNA and protein are expressed at high levels in tumors of Tax transgenic mice and tissues such as the submandibular gland and skeletal muscle, which express high levels of Tax mRNA [47]. Elevated levels of inflammatory cytokines (e.g., IL-1α, IL-1β, IL-6, tumor necrosis factor α, TGF-β1, IFN-γ, and IL-2) and major histocompatibility complex (MHC) genes were detected in the joints of env-pX transgenic mice, which develop inflammatory arthritis [48]. In primary tumors freshly isolated from Tax transgenic mice, the expression of NF-κB-inducible cytokines such as IL-6, IL-10, IL-15, and IFN-γ was elevated [49]. These findings suggest that in Tax transgenic mice, Tax constitutively activates NF-κB to enhance cell activation and inflammatory cytokine expression. The involvement of NF-κB activation in cell transformation, tumor development, and proliferation in Tax transgenic mice was demonstrated by sodium salicylate and prostaglandins, which inhibit NF-κB activity, inhibiting spontaneous proliferation of splenocytes in Tax transgenic mice. In addition, Tax-induced tumor cells resistant to radiation-induced apoptosis showed increased susceptibility to apoptosis in the presence of sodium salicylate and prostaglandin [49]. Mice treated with an antisense NF-κB oligodeoxynucleotide show rapid regression of transplanted Tax-transformed fibrosarcomas [50]. In contrast, transgenic mice with a Tax mutant defective in NF-κB activation fail to immortalize T cells and do not develop tumors [51]. In Tax transgenic mice, constitutive activation of NF-κB by Tax is involved in cell transformation, tumor growth, and resistance to apoptosis.

Tumors from Tax transgenic mice are reportedly characterized by mutations in the p53 tumor suppressor gene and functional inactivation of wild-type p53 protein [52]. Tax transgenic mice heterozygous for the p53 gene show more rapid tumor dissemination and increased mortality. Experiments crossing Tax transgenic mice with p53-deficient mice show little or no acceleration of early tumorigenesis, whereas mice heterozygous for p53 show markedly accelerated disease progression and death. These studies suggest that the functional inactivation of p53 by HTLV-1 Tax is unimportant for early tumorigenesis but contributes to late tumor progression [53]. IFN-γ-deficient Tax transgenic mice display accelerated tumor development, metastasis, and death [54]. IFN-γ-deficient Tax transgenic mice also display increased osteolytic bone lesions, soft tissue tumors, and osteoclast formation and activity [55]. The effect of osteoclast inhibition on tumorigenesis is indicated by double-transgenic mice for Tax, the osteoclast activator osteoprotegerin being protected from osteolytic bone disease, and the development of fewer soft tissue tumors. Similarly, osteoclast suppression by zoledronic acid attenuates bone and soft tissue tumors to prolong the survival of Tax transgenic mice [42].

Transgenic mice expressing HBZ under the control of the granzyme B promoter (Gzmb-HBZ) develop lymphoproliferative diseases such as tumors, splenomegaly, abnormal white blood cell numbers, and hypercalcemia [56]. Transgenic mice expressing HBZ under the control of the CD4 promoter generated T cell lymphoma and systemic inflammation [14]. HBZ transgenic mice have proliferating Foxp3^+^ Treg cells and effector/memory T cells. HBZ directly induces the transcription of Foxp3 in T cells, leading to their differentiation into Foxp3^+^ Treg cells. However, Treg function is disrupted by the interaction of HBZ with Foxp3 and NFAT. Because Foxp3^+^ T cells in HBZ transgenic mice have unstable Foxp3 expression, Foxp3^+^ T cells were transformed into Foxp3^−^ T cells with enhanced IFN-γ production [57]. HBZ transgenic mice lacking IFN-γ display reduced incidence of inflammation and lymphoma [58]. These findings implicate increased IFN-γ production in the development of inflammation and lymphoma in HBZ transgenic mice. HBZ transgenic mice display a higher expression of T cell immunoglobulin and ITIM domain (TIGIT, a co-suppressor molecule), and increased expression of IL-10 in TIGIT^+^ CD4^+^ cells [59]. HBZ was shown to interact with Grb2 and SHP-2-associated THEMIS, inhibiting suppressive signals from programmed death 1 and TIGIT to promote T cell proliferation [60]. Although IL-10 is an immunosuppressive cytokine, HBZ was shown to interact with both signal transducer and activator of transcription (STAT1) and STAT3, and modulate the IL-10/Janus kinase (JAK)/STAT signaling pathway to promote T cell proliferation [61]. Vaccination with recombinant vaccinia virus expressing HBZ induced HBZ-specific T cell responses, although several boosters were required. The transplantation of splenocytes from HBZ-immunized mice into HBZ transgenic mice’s increased survival, suggesting that anti-HBZ CTLs have a protective effect [62].

HBZ/Tax double-transgenic mice exhibit skin lesions and T cell lymphoma [63]. ATL stem cells (ATLSCs) were identified in Tax transgenic and HBZ transgenic mice. In Tax transgenic mice, c-kit^+^/CD38^−^/CD71^−^ cells were identified as an ATLSC candidate [64]. In HBZ transgenic mice, c-kit^+^/CD4^−^/CD8^−^ cells were identified as an ATLSC candidate [65].

## 3. Development of Severely Immunodeficient Mice

Humanized mice are severely immunodeficient mice into which human cells and tissues are engrafted. To engraft human cells into mice, murine immune cells must be defective or dysfunctional. Various severely immunodeficient mouse models have been developed (Table 1). SCID (severe combined immunodeficiency) mice are severely immunodeficient and lack functional T and B cells [66]. Because of nonsense mutations in the DNA-dependent protein kinase catalytic subunits (DNA-PKcs) encoded by *PRKDC* (protein kinase, DNA-activated, catalytic polypeptide), DNA non-homologous end-joining (a recombination pathway for DNA double-strand breaks required for VDJ recombination) is defective in T and B cell receptors [67]. SCID mice were engrafted with mature human T and B cells upon transplantation with human fetal liver hematopoietic cells, human fetal thymus, and human fetal lymph nodes [68]. SCID mice were also short-term engrafted with human peripheral blood lymphocytes (PBL) when inoculated intraperitoneally [69]. However, natural killer (NK) cells and macrophages, which are involved in innate immune responses, have normal functions in SCID mice [70]. SCID mice leak T and B cells as they age [71]. In addition, SCID mice are more sensitive to irradiation because of DNA repair abnormalities. NOD/SCID mice were generated by crossing SCID mice with non-obese diabetic (NOD) mice, which have reduced NK cell, dendritic cell (DC), and macrophage activity [72]. NOD mice have a 2-bp deletion in the *C5* gene, resulting in losses of functional C5 and membrane attack complex formation [73]. Mouse strains crossed with NOD mice, including NOD/SCID mice, inherit this defect from NOD mice. NOD/SCID mice display losses of T cell, B cell, and complement activity, and reduced NK cell, DC, and macrophage activity. In addition, NOD/SCID mice have an improved human PBMC engraftment rate compared with SCID mice [74]. However, NOD/SCID mice also display the characteristics of SCID mice, such as the leakage of T and B cells and high radiosensitivity. Furthermore, NOD/SCID mice develop thymic lymphoma with a high frequency and have a short lifespan. NOG (NOD/SCID/IL2Rγ^null^) and NSG (NOD/SCID/IL2Rγ^null^) mice were developed by crossing NOD/SCID mice with mice lacking the IL-2R γ chain, which are defective in NK cells [75,76]. The IL-2R γ chain in NOG mice harbors a mutation that allows cytokines to bind but not elicit signals, while the IL-2R γ chain in NSG mice is completely defective. NOG and NSG mice display deficient T cell, B cell, NK cell, and complement activity, and have impaired DC and macrophage functions. NOG and NSG mice do not develop T or B cell leakage, possibly because signals from the IL-2R γ chain are responsible for the differentiation and proliferation of T and B cells. NOG and NSG mice have a higher rate of human cell engraftment than NOD/SCID mice. Immunodeficient mice with phenotypes similar to NOG and NSG mice have also been developed. For example, NOJ (NOD/SCID/JAK3^null^) mice were developed by crossing NOD/SCID mice with mice lacking JAK3, a tyrosine kinase involved in downstream signaling of the IL-2R γ chain [77]. As an alternative to SCID mice, NRG (NOD/Rag1^null^/IL-2Rγ^null^) mice were developed by crossing mice deficient in recombination activating gene 1 (Rag1), a DNA recombinase essential for genetic reconstitution of T and B cell receptors [78].

## 4. Immunodeficient Mouse Models for HTLV-1 Research

Immunodeficient mice can be transplanted with HTLV-1-infected cell lines or patient-derived PBL. SCID mice depleted of NK cells via the administration of an anti-asialo GM-1 antibody formed tumors when transplanted with MT-2 cells, an HTLV-1-infected cell line [79]. Intraperitoneal inoculation of MT-2 cells also resulted in tumor formation in SCID mice [80]. PBL from patients with ATL or HAM were inoculated into SCID mice [81,82]. The characterization of the inoculated cells revealed that ATL-derived PBL developed lymphoblastic lymphoma, while HAM-derived PBL had superior proliferative potential compared with uninfected PBL. T cells immortalized via the gene transfer of *Tax* in vitro could not be maintained when transplanted into SCID mice [83]. Cells derived from Tax transgenic mice were maintained when transplanted into SCID mice, but the viable cells showed no detectable expression of Tax at the protein level and displayed constitutive NF-κB and Akt activity [41,84]. PDZ and LIM domain protein-2 (PDLIM2) is an E3 ligase that binds to the p65 subunit of NF-κB and STAT1, STAT3, and STAT4 in the nucleus, thereby promoting the degradation of these proteins and inhibiting NF-κB activation and inflammatory cytokine production [85,86,87]. PDLIM2 binds to Tax in the nucleus, allowing its degradation by the proteasome. Transplantation of cell lines transfected with Tax and PDLIM2 into SCID mice exhibited reduced tumorigenesis compared with transplantation of cell lines transfected with Tax alone [88]. Whether Tax is involved in cell proliferation and survival in vivo is inconclusive. ATL cells from patients with ATL cannot express Tax because of mutations or promoter methylation, suggesting that ATL cells eventually no longer require Tax for proliferation and survival [89]. HBZ-knockdown HTLV-1 cell lines show markedly reduced tumor formation and organ invasion when transplanted into NOG mice [90]. HBZ was shown to play an important role in the proliferation and survival of HTLV-1-infected T cells.

Immunodeficient mice have contributed to HTLV-1 research as an in vivo model to evaluate candidate molecules for ATL therapeutics. An ATL model has been developed in which HTLV-1-infected and ATL cell lines are transplanted subcutaneously or into the abdominal cavity of immunodeficient mice, allowing measurement of tumor formation and tumor invasion into various organs. Since then, numerous preclinical studies of candidate molecules for ATL therapeutics have been conducted using the ATL model (Table 2). The NF-κB inhibitors Bay 11-7082 [91] and dehydroxymethylpoxyquinomycin (DHMEQ) [92,93,94,95] inhibit tumor formation and invasion derived from HTLV-1-infected cell lines implanted in severely immunodeficient mice. Other drugs that inhibit NF-κB activation have also been used as single agents or in combination with other drugs to treat ATL tumors, including bortezomib (PS-341, a proteasome inhibitor) [96,97], ritonavir (an HIV protease inhibitor) [98], diarsenic trioxide (As_2_O_3_, which degrades Tax protein) [99], 17-dimethylaminoethylamino-17-demethoxygeldanamycin hydrochloride (an inhibitor of heat shock protein 90) [100], and 9-aminoacridine [101]. Histone deacetylase inhibitors (HDACi) induce histone hyperacetylation, resulting in chromatin remodeling and reactivation of transcriptional repressor genes, and have shown efficacy against a variety of cancers. The HDACi depsipeptide [102], LBH589 [103], and AR-42 [104] prolonged the survival of severely immunodeficient mice transplanted with HTLV-1-infected cell lines either as single agents or in combination with other drugs. The efficacy of apoptosis inhibitors and other inhibitors in the treatment of ATL has also been examined in an ATL model. ABT-737 (a small molecule inhibitor of Bcl-2, Bcl-X(L), and Bcl-w, which act in antiapoptosis) [105], fucoidan (a sulfated polysaccharide that inhibits survivin, an antiapoptotic protein) [106], the autophagy inhibitors chloroquine and hydroxychloroquine [107], the adenosine monophosphate-activated protein kinase (AMPK) inhibitor dorsomorphin [108], the importin (IPO) α/β1 inhibitor ivermectin [109], the selective JAK inhibitor ruxolitinib in combination with the Bcl-2/Bcl-xL inhibitor navitoclax [110], and the dual SYK/JAK inhibitor cerdulatinib [111] suppressed tumors derived from HTLV-1-infected cells transplanted into immunodeficient mice. Other agents with antitumor activity include incadronate (a bisphosphonate) [112], indole-3-carbinol (a naturally occurring component of cruciferous vegetables) [113], and the biphosphine cyclopalladium complex [Pd_2_(S^-^C_2_,N-dmpa)_2_(μ-dppe)Cl_2_] [114]. In addition, the carotenoid peridinin [115], bioactive plant polyphenol butein [116], and organic compound thymoquinone in combination with low concentrations of doxorubicin (Dox) [117] were shown to be effective in treating ATL using ATL models. ATL cells are characterized by invasion into organs and skin. AMD3100, a CXCR4 antagonist, inhibited lymphoma cell infiltration into liver and lung tissues in vivo in an ATL model [118].

The ATL model with immunodeficient mice is a superior system for evaluating drug activity against ATL cells. However, immunodeficient mice are deficient in host immune cells and cannot be used for experiments involving host immune responses, such as vaccines or immune checkpoint therapy. These experiments require humanized mice/human immune system mice—immunodeficient mice in which human immune cells are reconstituted.

## 5. Development of Human Immune System Mice

**Figure 1:**
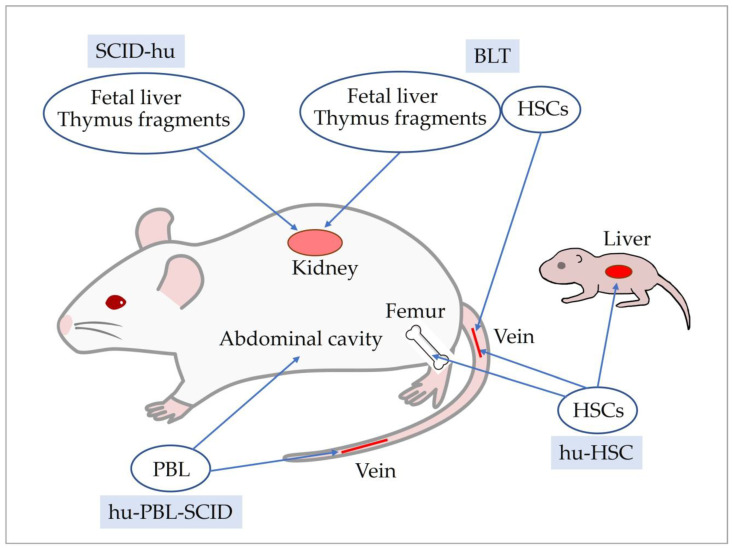
Generation of human immune system mouse models. Abbreviations: BTL, bone marrow liver thymus; PBL, peripheral blood lymphocyte; HSC, hematopoietic stem cell.

Several methods are available to reconstitute the human immune system in severely immunodeficient mice, and the humanized mice/human immune system (HIS) mice generated using each method have different characteristics (Figure 1). The simplest is a method for the huPBL-SCID mouse model, which is generated via intravenous or intraperitoneal injection of human PBL into SCID mice [69]. In huPBL-SCID mice, transplanted cells survive for approximately 3 weeks after transplantation. Thereafter, immune cells that react to host mouse cells, mainly effector T cells, and only a few B cells are detected in huPBL-SCID mice [119]. huPBL-SCID mice have low human immune cell viability and reconstitution and develop severe xenogeneic graft-versus-host disease, which can be reduced by utilizing NSG mice deficient in murine MHC class I and class II as hosts [120].

The SCID-hu mouse model is humanized mice in which human fetal thymus and liver are transplanted under the renal capsule of SCID mice [68]. SCID-hu mice display low reconstitution of human immune cells and short T cell survival. The bone marrow liver thymus (BLT) mouse model, a modification of the SCID-hu mouse model, is generated by transplanting autologous human hematopoietic fetal liver CD34^+^ cells into NOD/SCID mice after the engraftment of human fetal thymus and liver [121]. BLT mice display human T cells throughout the body for an extended period of time. In addition, repopulation of T cells, B cells, and DCs was observed in secondary lymphoid tissues. A significant advantage of BLT mice is that thymic transplantation induces the differentiation of human HLA-recognizing T cells, which can induce a human MHC-restricted immune response [122,123]. However, the BLT mouse model is difficult to create because the availability of human fetal tissue is ethically and legally restricted in many countries.

hu-HSC mouse models are humanized mice generated by transplanting human hematopoietic stem cells (HSCs) into severely immunodeficient mice. Variations in these models arise from differences in human HSCs used for transplantation and the method of transplantation. Two types of HSCs are used for transplantation: CD34^+^ cells and CD133^+^ cells [124,125,126]. CD133^+^ cells are more undifferentiated than CD34^+^ cells and can differentiate into neural and mesenchymal cells [127,128,129]. The transplantation of CD133^+^ cells may affect the microenvironment of the bone marrow, where immune cells differentiate and are maintained. The first transplantation method of HSCs involves irradiating or administering busulfan to severely immunodeficient mice to create space in the bone marrow niche. Mice are then humanized via intravenous injection or intramedullary inoculation of human HSCs. Intra-bone marrow injection allows for a smaller number of HSCs to be used [124]. The second method of HSC transplantation involves the inoculation of the liver of neonatal severely immunodeficient mice [125]. The advantages of this method are that the number of HSCs inoculated can be much lower than that in adult mice and humanization can be completed earlier. The characteristics of hu-HSC mice generated via these methods are similar, with reconstitution of B cells, DCs, macrophages, and NK cells by approximately 8 weeks after HSC transplantation, and T cells slightly later, by approximately 12 weeks after transplantation [124,130]. The reconstituted human immune cells are maintained for a long period of time.

## 6. Improvements in the hu-HSC Mice

Although the hu-HSC mouse model reconstitutes and maintains acquired and innate immune cells for a long time, it does not fully recapitulate human immune responses and has various issues. The most significant issue is that hu-HSC mice have weak acquired immune responses. T cells in hu-HSC mice cannot recognize human MHC (HLA) because they differentiate in the mouse thymus. Therefore, T cells cannot receive antigen stimulation from human antigen-presenting cells or elicit a human immune response. In B cells, antigen-specific IgM can be detected but antigen-specific IgG is rarely detected. It is speculated that the T-B interaction required for antibody class switching in B cells may be insufficient. To address these issues, HLA class II transgenic NSG [NSG Tg(HLA-DR4)] and NOG [NOG Tg(HLA-DR4)] mice were developed to display the induction of a B cell class switch [131,132]. NSG mice expressing HLA class I-A2 [NSG Tg(HLA-A2)] and NSG mice expressing HLA class I-A2 and human β2 microglobulin binding domain [NSG Tg(HLA-A2, B2M)] were also developed [133,134]. These mice develop functionally mature CD8^+^ T cells. hu-HSC mice have fewer innate immune cells, such as DCs, macrophages and NK cells, likely because of a deficiency of cytokines that allow these cells to differentiate and proliferate. The administration of cytokines or cytokine expression vectors to mice or cytokine-transgenic mice improved myeloid and NK cell reconstitution [135,136,137,138,139]. NOG mice have inadequate lymph node development due to a deficiency of the IL-2R γ chain. NOG-pRORγt-γc Tg mice have been developed in which the IL-2R γ chain gene is expressed in lymphoid tissue of NOG mice under the induction of the endogenous promoter of RORγt. Humanized NOG-pRORγt-γc Tg mice have normalized lymph node organogenesis and enhanced antigen-specific IgG responses [140].

## 7. Human Immune System Mouse Models for HTLV-1 Research

The clonality of HTLV-1-infected cells was examined in the huPBMC-NOG mouse model. Primary infection was established when HTLV-1-uninfected PBMCs were transplanted into NOG mice, which were subsequently inoculated with HTLV-1-infected MT-2 cells [141]. The administration of the reverse transcriptase inhibitors azidothymidine and tenofovir, which suppress de novo infection, to huPBMC-NOG mice prevented primary HTLV-1 infection. However, the administration of tenofovir one week after infection did not affect the proviral load (PVL) [141]. In the huPBMC-NOG mouse model, PBMCs from asymptomatic HTLV-1 carriers were transplanted into NOG mice; one month after infection, specific clones of HTLV-1-infected cells were selectively proliferating [142]. These results suggest that in the huPBMC-NOG mouse model, de novo infection dominates the early stages of HTLV-1 infection, while clonal proliferation dominates as infection progresses. 

An HTLV-1-infected hu-HSC mouse model was developed to characterize infected cells. HIS Rag2^−/−^γc^−/−^ mice were generated via the intrahepatic transplantation of human CD34^+^ cells into neonatal BALB/c Rag2^−/−^γc^−/−^ mice [143]. Several months after infecting HIS Rag2^−/−^γc^−/−^ mice with HTLV-1, hepatosplenomegaly, lymphadenopathy, and lymphoma/thymoma were observed in which Tax was detected [143]. IBMI-huNOG mice were generated via an intra-bone marrow injection (IBMI) of CD133^+^ cells into NOG mice [124]. HTLV-1 infection of IBMI-huNOG mice resulted in a rapid increase in peripheral CD4^+^ T cells and PVL. The mice developed little lymphoma, although an enlargement of the spleen and liver was observed. These leukemic signs were observed from the early stages of infection, and cells with a laminar nucleus morphologically similar to the flower cells observed in patients with ATL were detected in peripheral blood 4 to 5 months post-infection. Moreover, HTLV-1-infected IBMI-huNOG mice were cytokineemic, similar to patients with ATL [124]. A high clonality of cell populations was detected in mice infected for as long as 18 or 23 weeks [124]. A significant feature of HTLV-1-infected IBMI-huNOG mice was the detection of Tax-specific CTLs and the production of HTLV-1-specific IgG. HIS NSG mice were generated via the engraftment of CD34^+^CD38^−^lin^−^ HSCs into the liver of neonatal NSG mice [125]. HTLV-1 infection of HIS NSG mice resulted in a rapid increase in CD4^+^ T cells in peripheral blood and lymphoid tissues. CD4^+^ T cells displayed phenotypes of effector memory cells and Th1 cells [125]. HTLV-1-infected HIS NSG mice generated by another group also developed lymphoproliferative transformation by 3 weeks post-infection, which progressed to terminal lymphoproliferative disease by 6–8 weeks (median survival, 5–6 weeks) post-infection [126]. HTLV-1-infected cells in these mice proliferated oligoclonally. These mice also developed osteolytic bone lesions.

In the hu-HSC mouse model, mutant HTLV-1 can be transmitted to mice by HTLV-1 transgenic cells with mutations in the HTLV-1 gene as the source of infection. To investigate the in vivo contribution of the Tax PDZ domain-binding motif (PBM) to the lymphocyte proliferation process, cells transfected with HTLV-1 gene harboring a deletion of the Tax PBM were generated [144]. hu-HSC mice inoculated with these cells for HTLV-1 infection showed reduced frequencies of activated CD4^+^ T cells in the peripheral blood and spleen compared with mice inoculated with wild-type transfected cells. These results suggest that the PBM of Tax is required for sustained proliferation of HTLV-1-infected T cells. To evaluate the role of HBZ in ATL-associated bone destruction, hu-HSC mice were infected with HTLV-1 lacking HBZ. Compared with wild-type HTLV-1-infected mice, HBZ-deficient HTLV-1-infected mice display a slight delay in lymphoproliferative disease and a significant reduction in disease-related bone loss [126]. An enhancer inside the virus was found in the *Tax* gene near the 3′ LTR of HTLV-1. To investigate whether this enhancer affects disease development, HTLV-1-infected cells carrying a mutant enhancer virus (mEnhancer) were transplanted into hu-HSC mice and evaluated. In mEnhancer-infected hu-HSC mice, the enhancer did not affect disease progression, PVL, or viral gene expression [145].

HTLV-1-infected hu-HSC mouse models are currently being evaluated for drugs that target HTLV-1-infected cells (Table 3). Truncated pseudomonas exotoxin 38 (PE38) fused with the CCR4 ligand CCL17/thymus and activation-regulated chemokine (TARC) was evaluated to selectively eliminate HTLV-1-infected cells [146]. Full-length pseudomonas exotoxin is known to bind specifically to CD91 on the cell surface via its CD91-binding domain and to subsequently be translocated intracellularly by endocytosis, causing cytotoxic effects dependent on the intracellular expression of furin, a protein convertase [147]. TARC-PE38 replaces the CD91-binding domain of PE with TARC, thereby exerting cytotoxic effects on CCR4-expressing cells. In HTLV-1-infected hu-HSC mice, TARC-PE38 markedly inhibits the proliferation of HTLV-1-infected human CD4^+^CD25^+^ cells and CD4^+^CD25^+^CCR4^+^ cells, and reduces PVL in PBMC [146]. HTLV-1-infected CD4^+^ T cells secrete leukotriene B4 (LTB4), a potent chemoattractant [148]. The inhibition of LTB4 secretion by MK886, an inhibitor of the 5-lipoxygenase (5-LO) cofactor FLAP (5-LO activating protein), in an HTLV-1-infected hu-HSC mouse model reduces both HTLV-1 PVL and the number of infected clones [148]. Recombinant vesicular stomatitis virus (VSV) has also been used to target HTLV-1-infected cells. VSV infects a variety of cell types in vitro, produces many progeny virions, and induces cytolysis of infected cells. VSVΔG-GL and VSVΔG-NP were prepared by replacing VSV-G (the envelope glycoprotein of VSV) with GLUT1 and NRP1, which HTLV-1 binds as receptors [149]. In the HTLV-1-infected hu-HSC mouse model, VSVΔG-NP efficiently prevented HTLV-1-induced leukocytosis in the periphery and eliminated HTLV-1-infected Env-expressing cells in bone marrow and the spleen [149]. The transcription factor myocyte enhancer factor (MEF)-2 family member MEF-2C was enriched in the 3′ LTR of HTLV-1 and observed to bind HBZ in this region, along with its cofactors Menin and JunD. Chemical inhibition of the MEF-2 protein reduced PVL in the HTLV-1-infected hu-HSC mouse model [150]. Protein arginine methyltransferase 5 (PRMT5), a type II PRMT enzyme, is directly involved in the pathogenesis of several different lymphomas through transcriptional regulation of related oncogenes [151]. EPZ015666, an inhibitor of PRMT5, enhanced survival of HIS NSG mice inoculated with HTLV-1-producing cells [152]. 

## 8. Advantages and Disadvantages of Each Mouse Model in the Development of ATL Therapeutics/Prophylaxis

**Figure 2:**
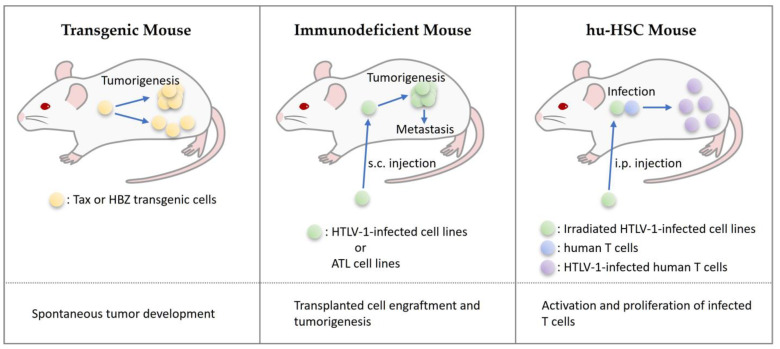
Characteristics of mouse models used in ATL treatment studies. Abbreviations: s.c., subcutaneous; i.p., intraperitoneal.

Because Tax and HBZ transgenic mice spontaneously develop tumors, they can be used to investigate therapeutic agents for ATL (Figure 2). In addition, cells derived from these transgenic mice can be transferred into wild-type mice immunized with Tax or HBZ to provide a vaccine model against Tax or HBZ. The disadvantages of these models include the late onset of tumors and variability in the frequency of tumor onset. In addition, this tumor model is based on the overexpression of Tax and HBZ, which is different from actual tumorigenesis of HTLV-1-infected cells.

Immunodeficient mice have been developed as tumor and metastasis models of HTLV-1-infected and ATL cell lines. This model has been used in many studies of ATL therapeutics because of its cost and simplicity. However, the primary limitation is that the transplanted cells are from cell lines, which have altered characteristics compared with original HTLV-1-infected cells and ATL cells. In addition, these mice do not have an immune system and cannot be used to study vaccines that induce CTLs or antibodies.

hu-HSC mice will be used to develop prophylaxis for ATL that targets infected cells during the early stages of HTLV-1 infection. HTLV-1-infected T cells in these mice are activated and strongly induced to proliferate, meaning their characteristics are more similar to ATL cells than infected cells of carriers. The disadvantage of this model is that the humanization of mice is time-consuming and labor-intensive, and mice develop graft-versus-host disease and die early. In addition, currently available hu-HSC mice have weak human-acquired immunity, making it difficult to use them for research on vaccines that induce CTLs and antibodies.

## 9. Conclusions

Transgenic mice for HTLV-1 *Tax* and *HBZ* are useful tools for understanding the role of these genes in the pathogenesis of HTLV-1-related leukemia and lymphoma (ATL). Severely immunodeficient mice enable the transplantation of ATL cell lines, advancing the development of ATL therapeutics. Humanized mice with reconstructed human immune systems (HIS mice) have been used, for example, to develop preventive drugs targeting HTLV-1-infected cells. Current HIS mice do not fully recapitulate the human immune system. Therefore, next-generation humanized mice with different reconstructed immune cells are currently being developed to improve the modeling of the human immune system in mice. It is necessary to select the most appropriate next-generation HIS mice for HTLV-1 research. In the future, animal models for HTLV-1 infection and ATL using next-generation HIS mice are expected to be developed for various purposes, such as elucidating the pathogenesis of ATL, developing methods to prevent the onset of ATL, and developing vaccines against HTLV-1 infection and drugs to prevent or treat ATL.

## Figures and Tables

**Table 1:** Characteristics of severely immunodeficient mice.

Strain	Genotype	Characteristics	Limitation	Reference
SCID	*Prkdc^SCID^*	T cell and B cell defect	T cell and B cell leakage Radiosensitive	[66]
NOD/SCID	NOD/*Prkdc^SCID^*	T cell and B cell defect Decreased NK cell, DC and macrophage activity Functional C5 complement deficiency	T cell and B cell leakage Radiosensitive Spontaneous lymphoma	[72]
NOG	NOD/*Prkdc^SCID^*/*Il2rg^null^*	T cell, B cell and NK cell defect Decreased DC and macrophage activity Functional C5 complement deficiency	Radiosensitive	[75]
NSG	NOD/*Prkdc^SCID^*/*Il2rg^null^*	T cell, B cell and NK cell defect Decreased DC and macrophage activity Functional C5 complement deficiency	Radiosensitive	[76]
NOJ	NOD/*Prkdc^SCID^*/*JAK3^null^*	T cell, B cell and NK cell defect Decreased DC and macrophage activity Functional C5 complement deficiency	Radiosensitive	[77]
NRG	NOD/*Rag1^null^*/*Il2rg^null^*	T cell, B cell and NK cell defect Decreased DC and macrophage activity Functional C5 complement deficiency	-	[78]

**Table 2:** ATL therapeutic candidates evaluated in severely immunodeficient mice.

Candidates	Target/Function	Mouse	Efficacy	Reference
Bay 11-7082	NF-κB inhibitor	NOG mouse	Inhibits tumor growth and invasion	[91]
DHMEQ	NF-κB inhibitor	NK(-)SCID mouse SCID mouse	Inhibits tumor growth and invasion	[92,93,94,95]
Ritonavir	HIV protease inhibitor	NOG mouse	Inhibits tumor growth and invasion	[98]
Fucoidan	Survivin inhibitor	SCID mouse	Partially inhibits tumor growth	[106]
Incadronate	Mevalonate pathway inhibitor	SCID mouse	Reduces tumor formation	[112]
PS-341	Proteasome inhibitor	SCID mouse	Inhibits tumor growth	[96]
PS-341 Zoledronic acid	Proteasome inhibitor Osteoclast inhibitor	NOD/SCID mouse	Inhibits tumor growth	[97]
9-aminoacridine (9AA) Campath-1H	Increase p53 transcription activity NF-κB activation inhibitor Humanized anti-CD52 antibody	NOD/SCID mouse	Inhibits tumor growth Extends survival	[101]
Depsipeptide Daclizumab	HDAC inhibitor Anti-IL-2Rα antibody	NOD/SCID mouse	Extends survival	[102]
LBH589	HDAC inhibitor	SCID mouse	Induces tumor cell apoptosis Extends survival	[103]
AR-42	HDAC inhibitor	NOD/SCID mouse	Extends survival	[104]
ABT-737	Bcl-2, Bcl-X(L), and Bcl-w inhibitor	SCID mouse	Inhibits tumor growth	[105]
17-DMAG	HSP90 inhibitor	NOG mouse	Inhibits tumor invasion Extends survival	[100]
As(2)O(3) IFN-α	Proteolysis of Tax Antiviral	SCID mouse	Inhibits tumor cell immortality	[99]
C7a	Antitumor effect	NSG mouse	Extends survival	[114]
Indole-3-carbinol	Antitumor effect	SCID mouse	Inhibits tumor growth	[113]
AMD3100	CXCR4 antagonist	SCID mouse	Inhibits tumor cell infiltration into liver and lung tissue	[118]
Chloroquine Hydroxychloroquine	Autophagy inhibitor	NOG mouse	Inhibits tumor growth Extends survival	[107]
Dorsomorphin	AMPK inhibitor	NOD/SCID mouse	Inhibits tumor growth	[108]
Ivermectin	IPOα/β1 inhibitor	SCID mouse	Inhibits tumor growth	[109]
Ruxolitinib Navitoclax	JAK inhibitor Bcl-2/Bcl-xL inhibitor	NSG mouse	Inhibits tumor growth	[110]
Cerdulatinib	Dual SYK/JAK inhibitor	SCID mouse	Inhibits tumor growth	[111]
Peridinin	Antitumor effect	SCID mouse	Inhibits tumor growth	[115]
Butein	Antitumor effect	SCID mouse	Inhibits tumor growth	[116]
Thymoquinone Doxorubicin	Antitumor effect Anticancer drug	NOD/SCID mouse	Inhibits tumor growth	[117]

**Table 3:** ATL therapeutic candidates evaluated in hu-HSC mice.

Candidates	Target/Function	Mouse	Efficacy	Reference
TARC-PE38	CCR4	HIS NOJ mouse	Eliminate HTLV-1-infected cells	[146]
MK886	LTB4 secretion inhibitor	HIS NSG-HLA-A2/HDD mouse	Reduce both PVL and the number of infected clones	[148]
VSVΔG-GL VSVΔG-NP	GLUT1 NRP1	HIS NOJ mouse	Eliminate HTLV-1-infected Env-expressing cells	[149]
MC1568	HDAC inhibitor (MEF-2 inhibitor)	HIS NOG mouse	Reduce PVL	[150]
EPZ015666	PRMT5 inhibitor	HIS NSG mouse	Extends survival	[152]

## Data Availability

Not applicable.
